# Continuous release of oregano oil effectively and safely controls *Varroa destructor* infestations in honey bee colonies in a northern climate

**DOI:** 10.1007/s10493-017-0157-3

**Published:** 2017-07-26

**Authors:** Qodratollah Sabahi, Hanan Gashout, Paul G. Kelly, Ernesto Guzman-Novoa

**Affiliations:** 1 School of Environmental Sciences, University of Guelph, 50 Stone Road East, Guelph, ON N1G 2W1 Canada; 2 Plant Protection Department, University of Tehran, Karaj, 31587-77871 Iran; 3 Plant Protection Department, Alfateh University, P.O. Box 13538, Tripoli, Libya

**Keywords:** *Varroa destructor*, *Apis mellifera*, Oxalic acid, Oregano oil, Clove oil, Delivery methods

## Abstract

The ectoparasitic mite *Varroa destructor* is responsible for the death of millions of honey bee (*Apis mellifera*) colonies worldwide. Testing potential miticide compounds with different delivery methods that effectively control *V. destructor* and have low toxicity for honey bees is crucial to manage this parasite in hives. We determined the varroacide efficacy of three natural compounds delivered to hives with three application methods over a 4-week period. Oxalic acid in a sucrose solution was applied impregnated in cardboard (T1). A mixture of oregano and clove oils in an ethanol-gelatin solution was applied impregnated in absorbent pads (T2). Oregano oil alone was delivered using electric vaporizers (T3) to test the hypothesis that continuous release of miticides increases the varroacidal efficacy of essential oils. The varroa mite control rates for treatments T1–T3 were 76.5 ± 7.11, 57.8 ± 12.79 and 97.4 ± 0.68%, respectively, and there were no differences for bee mortality between control and treatments 1 and 3. Additionally, most mites were killed in the first 2 weeks in T3 colonies compared to the last 2 weeks in colonies of the other treatments. These results demonstrate the importance of continuously releasing natural miticides to achieve safe and high rates of mite control in hives. They also show that oregano oil may be an effective miticide against *V. destructor* infestations in colonies.

## Introduction

The ectoparasitic mite *Varroa destructor* (Acari: Varroidae) is the most serious health problem for the honey bee, *Apis mellifera* (Hymenoptera: Apidae). This parasite is responsible for the death of millions of honey bee colonies worldwide, particularly in North America (Currie et al. 2010; Guzman-Novoa et al. 2010; van Engelsdorp et al. 2010; Guzman-Novoa 2016). *Varroa destructor* feeds on the hemolymph of brood and adult honey bees, inhibits their immune responses (Yang and Cox-Foster 2007; Koleoglu et al. 2017) and can transmit viral diseases, which in turn may lead to bee mortality (Martin 2001; Kevan et al. 2006; Emsen et al. 2015; Anguiano-Baez et al. 2016). If mite infestations are not controlled, colonies typically do not survive more than two years (De Jong 1997; Le Conte et al. 2010). Additionally, relatively low varroa mite infestations result in substantial reductions of honey yields of colonies located in both cold and warm climates (Currie and Gatien 2006; Medina-Flores et al. 2011; Emsen et al. 2014), but if treated with miticides, colonies produce more honey than untreated colonies (Arechavaleta-Velasco and Guzmán-Novoa 2000). However, the frequent use of synthetic miticides to control *V. destructor* infestations has resulted in mites developing resistance to many of the chemical components of these products (Milani 1999; Sammataro et al. 2005). The excessive or improper use of synthetic miticides has also caused contamination of hive products, which may pose a health threat for human consumers or for the bees in the hive (Wallner 1999; Bogdanov 2006). Therefore, the identification of new compounds with high miticidal activity and low toxicity to honey bees that at the same time do not leave harmful chemical residues in hive products is an important task for researchers (Rosenkranz et al. 2010).

Compounds of natural origin such as organic acids and essential oils are potential miticide candidates for use in hives (Imdorf et al. 1999; Fassbinder et al. 2002). It is well known that several essential oils and components of these substances exhibit miticidal activity against *V. destructor* (Imdorf et al. 1999) although not many of them, with the exemption of thymol, have been tested at the hive level. Therefore, to generate additional commercial formulations of natural miticides for the beekeeping industry, it is critical that essential oils that have shown high miticidal activity in laboratory trials, such as oregano and clove oil (Sammataro et al. 1998; Lindberg et al. 2000; Gashout and Guzman-Novoa 2009), are tested in hives.

A major problem evidenced in trials conducted to evaluate essential oils as miticides in hives, is that results are not always consistent, showing great variability between studies, seasons and localities (Calderone and Spivak 1995; Calderone et al. 1997; Imdorf et al. 1999; Emsen et al. 2007). Similarly, organic acids such as oxalic acid have shown varying degrees of mite control and are affected by numerous variables (Nanetti et al. 2003; Bacandritsos et al. 2007). One of the factors causing this variability is the pattern of climatic conditions influenced by ambient temperature and relative humidity. These variables can affect some properties of organic acids and essential oils such as their rate of evaporation, which in turn can affect the exposure of the mites to these products, which is crucial for successful mite control (Imdorf et al. 1999; Skinner et al. 2001). Therefore, developing effective delivery systems for releasing organic acids and essential oils continuously at a constant dose for an extended period regardless of environmental conditions, as well as determining the appropriate time of application (Currie and Gatien; 2006; Emsen et al. 2007; Melathopoulos et al. 2010), are important steps for using them practically in hives. However, few studies have been conducted to develop and test effective delivery systems of natural miticides in hives, which remains to be the greatest obstacle to consistently achieve high varroacidal efficacy of natural formulations. It is thus important to analyze the effect of multiple delivery systems intended for continuous miticide release and differing rates of miticide release, and to assess how these modes of release affect the miticide efficacy and safety.

In this study we evaluated the efficacy of one organic acid and two essential oils applied in hives with three delivery systems during early fall, for the control of *V. destructor* infestations of honey bee colonies. We also tested the hypothesis that the continuous release of miticides in hives increases the varroacidal efficacy of essential oils.

## Materials and methods

The trials were conducted in hives of the University of Guelph’s Honey Bee Research Centre in Guelph, ON, Canada. The level of varroa mite infestation of colonies headed by Buckfast queens and housed in Langstroth hives was determined by measuring their rate of mite drop before starting experimental treatments. The hives were equipped with screened bottom boards covered with 3 mm mesh hardware cloth that were used in conjunction with manila file folders coated with Crisco vegetable shortening, commonly referred to as sticky papers, placed underneath the cloth to capture falling mites and determine their daily fall rate. Twenty-four colonies were selected for having similar mite infestation rates and were moved to a single apiary where they were equalized to contain similar amounts of brood (700 ± 55 cm^2^), and adult bee population (ca. nine frames covered with bees). Groups of six colonies were randomly assigned to four treatment groups. The average daily rate of mite fall per hive before the beginning of experiments was 4.90 ± 0.58 mites, which did not significantly differ between treatment groups (F_3,92_ = 0.807, *p* = 0.49).

**Fig. 1:**
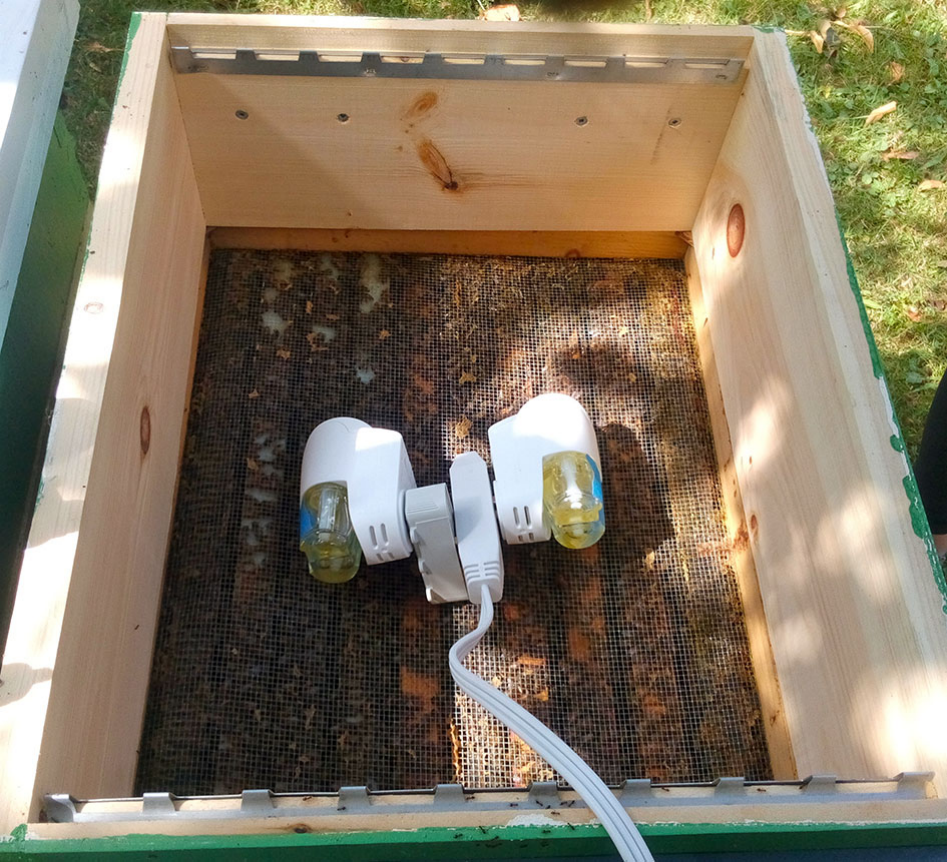
Electric vaporizers containing oregano oil installed above the brood chamber of honey bee colonies

The trials started on September 15, 2016 and the experimental colonies were treated weekly over 4 weeks with three formulations. The treatment delivery systems (carriers and solvents) of the formulations tested were chosen to provide either slow, rapid or continuous miticide release, based on experiments conducted to estimate rates and length of formulation release in hives in the same locality and season where experiments were conducted (Gashout 2008). Treatment 1, intended for slow miticide release, consisted of a 2% oxalic acid solution dissolved in a syrup made up of 65% sucrose and 35% H_2_O (w:v). A corrugated cardboard piece (30 × 23 × 0.5 cm) was soaked with 250 mL of the solution and placed on top of the brood chamber frames of each hive. Therefore, each colony received approximately 5 g of oxalic acid as active ingredient with each application. Treatment 2, intended for rapid miticide release, was a 7% solution of a mixture of oregano oil and clove oil (Sigma^®^, Missasauga, ON, Canada) in equal proportions. The solvent was prepared with 15 parts of 96% ethanol, 15 parts of H_2_O, two parts of food-grade mineral oil and one part of food-grade gelatin (Knox^®^, Tree House Foods, Winona, ON, Canada). Then, 30 ml of this solution were used to impregnate each of three 15 × 9 × 0.3 cm absorbent pads (Dri-Loc^®^, Cryovac, Elmwood, NJ, USA), which were applied in hives as above, as recommended by Gashout (2008), who found promising miticidal results for thymol with this delivery system. Each of these three pads contained approximately 1 g of each essential oil. Treatment 3, intended for continuous miticide release, was oregano oil (Sigma) in the 20 ml containers of two electric vaporizers (Febreze^®^ SY-982, China). The vaporizers with the oregano oil were placed above a 3 mm mesh hardware cloth of the dimensions of the hive on the top of the brood chamber frames, and a shallow super was placed above them to house them (Fig. 1). The vaporizers were plugged to an AC electric supply (120 V) for resistance heat, thereby allowing for evaporation of the oregano oil. The use of two vaporizers was decided based on results of preliminary evaporation trials that resulted in an average rate of release of 0.99 ± 0.4 g of oregano oil/day with two evaporators in a hive, and also from data indicating that about 0.85 g of oregano oil/day/hive would provide >60% mite control (Gashout 2008). To calculate the rate of evaporation of oregano oil, the vaporizer containers were weighed empty and filled with the essential oil at the beginning of trials to calculate the net weight, by subtracting the tare weight from the gross weight. Then, they were weighed again twice, every 2 weeks. Treatment 3 was intended to test the hypothesis that a continuous rate of evaporation of an essential oil at a constant dose would provide a high level of mite control. Treatment 4 was the control, colonies that did not receive any treatment.

To assess treatment efficacy and rate of mite fall, sticky papers were placed underneath the screen of the hives’ bottom boards to capture falling mites. The papers were collected twice a week and mites on them counted and divided by 3 or 4, to obtain an average rate of mites dropped per day. After 4 weeks of trials, two plastic strips containing amitraz (Apivar^®^, Véto-Pharma, Villebon-sur-Yvette, France) were placed in each hive as finisher treatment. The treatment was applied as indicated by the product label instructions. The efficacy of each treatment was determined using the following equation:

$${\text{Efficacy}}\left( \% \right) =\Sigma \left[ {{\text{N}}_{1} /\left( {{\text{N}}_{1} + {\text{N}}_{2} } \right)} \right] \times 100,$$

where, N_1_ is the number of mites that fell during the 4 weeks of test treatments, and N_2_ is the number of mites that fell during the final treatment with amitraz.

To find out what proportion of mites were killed by the treatments each week throughout the treatment period, relative mite-fall weekly rates (MWR) were calculated for each treatment as follows:

$${\text{MWR}} = \left[ {({\text{a}} + {\text{b}})/{\text{c}}} \right] \times 100,$$

where, a, is the number of mites that fell during the first observation of a week, b, is the number of mites that fell during the second observation of the same week, and c, is the total number of mites that fell during the 4 weeks of treatment.

To evaluate the effect of compounds on bee mortality, we installed Todd dead bee traps (Gary 1960) at the entrance of hives and counted dead bees twice a week during the trial’s 4-week period. Climatic data including ambient temperature, as well as relative humidity, were obtained from the Guelph Turfgrass Institute. The weather station is located 1.5 km from the Honey Bee Research Centre.

### Statistical analysis

Data on percent efficacy were arcsine-square root transformed to reduce variance heterogeneity and were subjected to analyses of variance (ANOVA). Data on bee mortality were log transformed before analyzing them by ANOVA. When significance was detected, means were separated with least significant difference (LSD) tests at α = 0.05. The Pearson correlation test was used to analyze the relationship between mite fall data and climatic variables. Paired t-tests were used for comparing oregano oil evaporation rates, as well as rates of mites fell per day at two intervals for treatment 3. SPSS v.22 software was used for analyzing the data (IBM 2013).

## Results

### Miticide efficacy

**Fig. 2:**
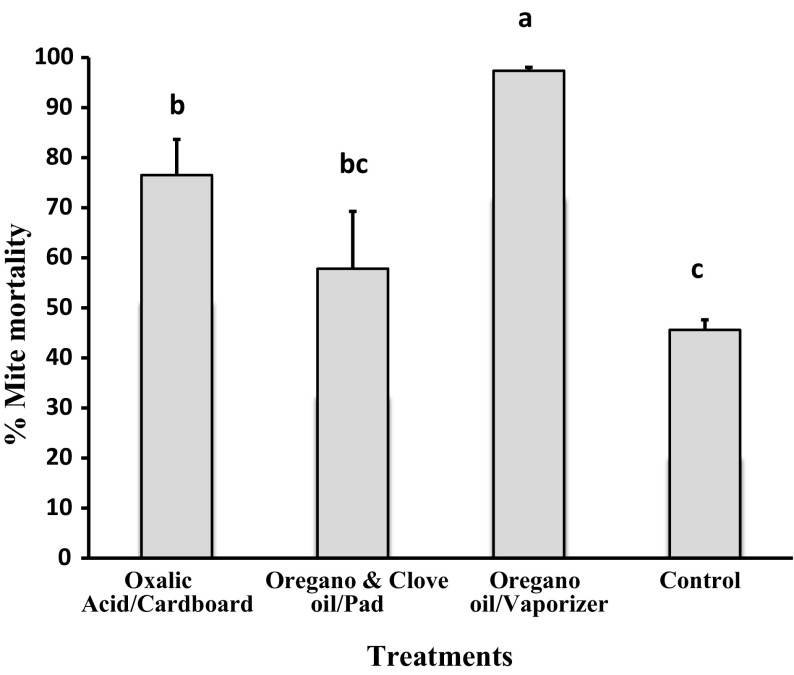
Mean varroa mite control efficacy (% ± SE) of three natural treatments in hives. Oxalic acid in a sugar solution was applied impregnated in cardboard, a mixture of oregano and clove oils was applied embedded in absorbent pads, whereas oregano oil alone was delivered with electric vaporizers. *Different letters* indicate significant differences of means based on analysis of variance and LSD tests of arcsine-square root transformed data. Non-transformed values are depicted

The efficacy rates for varroa mite control varied significantly among treatments (F_3,20_ = 11.718, *p* < 0.0001). Treatment 3 (oregano oil in vaporizers) had the highest efficacy rate (97.4 ± 0.68%), which was significantly higher than those of the other treatments. Conversely, the lowest efficacy rate was observed for treatment 2 (mixture of oregano and clove oils in pads), with 57.8 ± 12.79%, which did not differ with the control treatment. Treatment 1 (oxalic acid in cardboard) had a varroacidal efficacy rate of 76.5 ± 7.11%, which was significantly higher than that of control colonies (Fig. 2).

### Mite fall weekly rates (MWR)

**Fig. 3:**
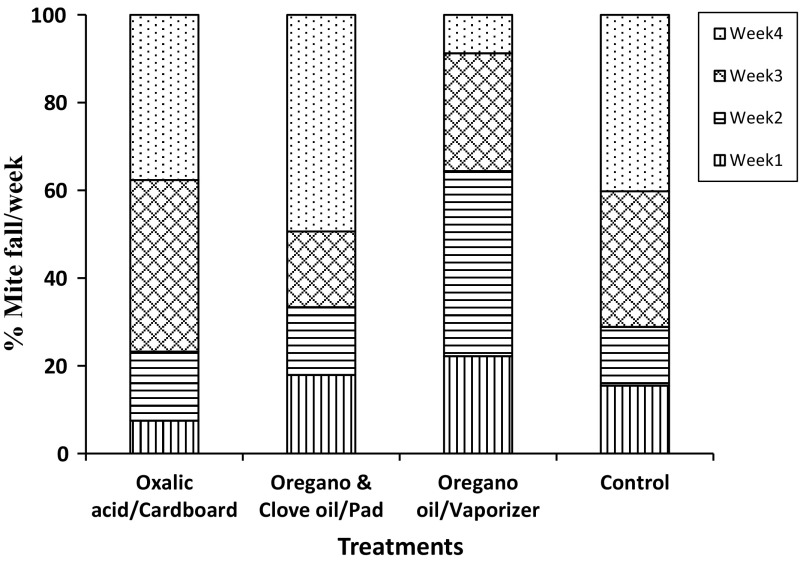
Mean weekly percent rates (MWR) of varroa mite fall for three natural treatments during 4 weeks in hives. Oxalic acid in a sugar solution was applied impregnated in cardboard, a mixture of oregano and clove oils was applied embedded in absorbent pads, whereas oregano oil alone was delivered with electric vaporizers

MWR were relatively higher for all treatments during weeks 3 and 4, including the control (>67%), than during weeks 1 and 2, except for treatment 3. Treatment 3 showed the highest proportion of mites killed during weeks 1 and 2 (65%). The most dramatic differences between treatments for MWR were observed during the last week, with treatments 1 and 2 having between 38 and 50% of the mites killed versus less than 9% for treatment 3 (Fig. 3).

### Miticide evaporation from vaporizers and mite fall

**Fig. 4:**
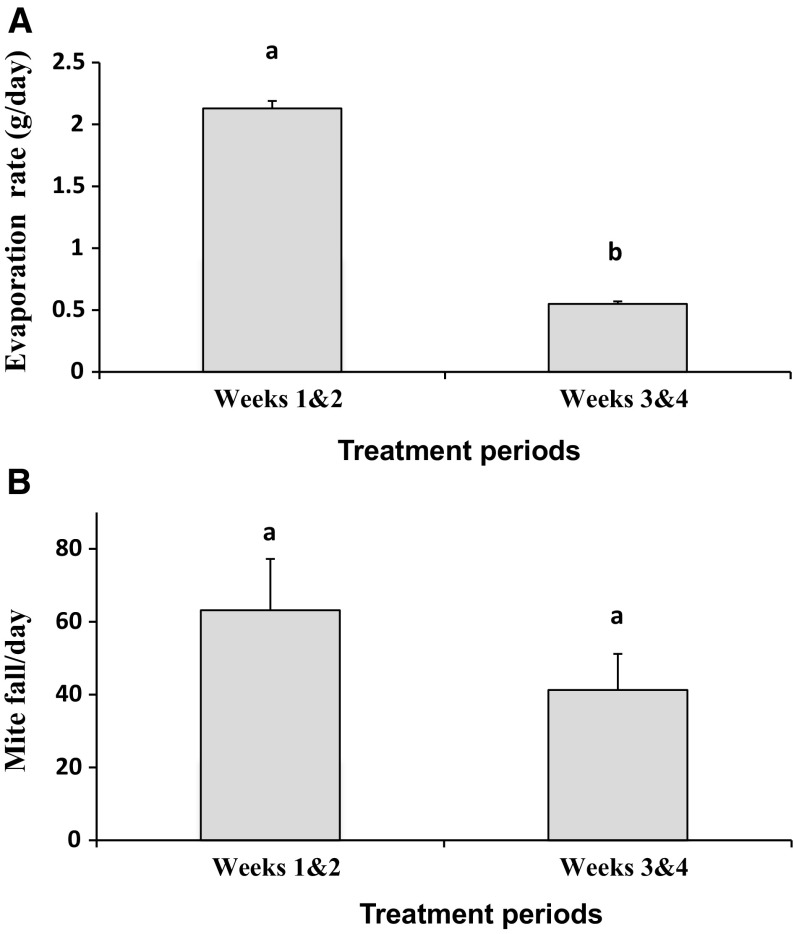
**A** Mean daily amount of evaporated oregano oil (g ± SE) from two electric vaporizers at two biweekly periods in hives. **B** Mean number of mites fell per day ± SE per treated colony at two biweekly periods. *Different letters* indicate significant differences of means based on paired sample t-tests

The evaporation rate of oregano oil from the electric vaporizers was significantly higher during the first period (first 2 weeks) than during the second period (last 2 weeks) of treatments (t_11_ = 33.54, *p* < 0.0001). In fact, the rate during the first period (2.13 ± 0.06 g/day) was almost 4× higher than that observed during the second period (0.55 ± 0.02 g/day; Fig. 4A). Despite this large difference in oregano oil evaporation rates between periods, the rates of mite fall per day between the same periods for the same treatment (63.17 ± 14.07 vs. 41.28 ± 9.91 mites/day) did not differ (t_11_ = 1.214, *p* = 0.25; Fig. 4B).

### Mite fall and climatic variables

No significant correlations were found between average ambient temperature and mite fall except for treatment 1 (r = −0.46, n = 28, *p* = 0.015). There was a correlation between average relative humidity and mite fall for treatment 1 (r = 0.42, n = 28, *p* = 0.03) too. No other significant correlations were found between climatic variables and mite fall.

### Worker bee mortality

**Fig. 5:**
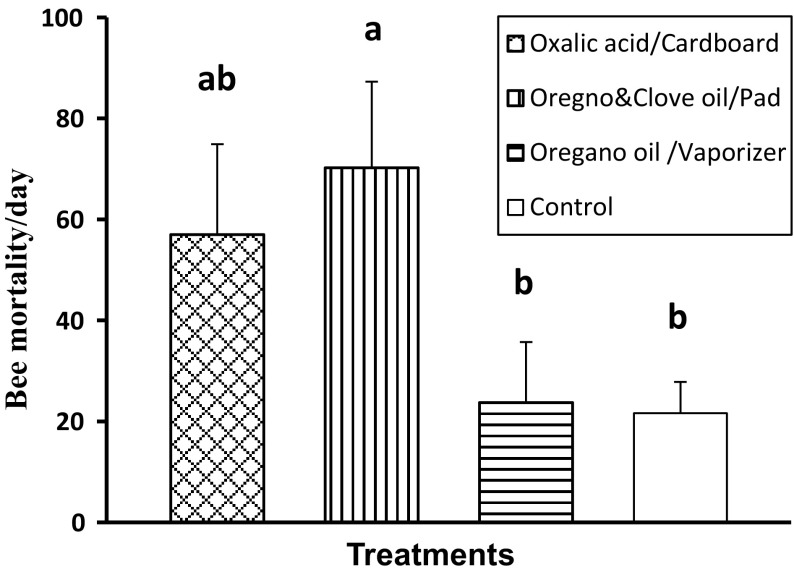
Mean number of worker bee deaths per day per colony ± SE following three natural treatments. *Different letters* indicate significant differences of means based on analysis of variance and LSD tests of log transformed data. Non-transformed values are depicted

The highest and lowest average number of worker bee deaths per day were found for treatments 2 (combination of essential oils in pad) and control (70.22 ± 17.05 and 21.62 ± 6.20%, respectively), which differed significantly from each other (F_3,28_ = 2.96, *p* < 0.05). However, treatments 1 (oxalic acid in cardboard) and 3 (oregano oil in vaporizers) were not different from control for bee mortality (Fig. 5).

## Discussion

The combination of various natural miticidal compounds and delivery systems used in this study showed considerable variability in their efficacy for controlling varroa mites during fall at the hive level. Our results demonstrate that oregano oil delivered with electric vaporizers that continuously released oil vapors in the hives can achieve a high level of varroa mite control (>97% efficacy). Conversely, delivery carriers designed to rapidly release miticides diluted in solvents (e.g. ethanol solutions in pads), yielded the lowest rate of mite control (<58%). Therefore, it is concluded that the efficacy of natural miticides in hives, is highly dependent on the delivery systems used.

Our study showed that an oxalic acid sucrose solution delivered in cardboard on multiple occasions, may provide a relatively high miticide efficacy of nearly 80%, which ranked this treatment as the second most efficacious formulation in the trials. Numerous studies have reported that oxalic acid is an effective organic compound for controlling varroa mite infestations in hives, particularly when brood is absent (reviewed in Rosenkranz et al. 2010). For example, Charrière and Imdorf (2002) showed that trickling sugar syrup containing 37 g of oxalic acid/L onto the bees resulted in effective control of varroa mites (>99%). Similarly, Coffey and Breen (2016) reported 81.5% mite control efficacy when using the trickling method to apply oxalic acid in hives. However, several researchers have pointed out the need for more than one treatment in colonies that have brood (Mutinelli et al. 1997; Bahreini 2003; Giovenazzo and Dubreuil 2011). In fact, Emsen et al. (2007) treated colonies containing brood with oxalic acid using the trickling method during early fall and in the same location where this study was conducted, and they found an average rate of varroa mite control below 40%. More recently, in a study aimed at providing high varroa mite control in colonies containing brood, Maggi et al. (2016) used cellulose strips embedded with 10 g of oxalic acid that were hung between hive frames and were able to achieve 93.1% mite control. It is clear then, that constant release of oxalic acid is needed when there is brood in honey bee colonies. Using corrugated cardboard as a vehicle to deliver oxalic acid in hives as in our study, is practical, simple, and inexpensive, but the level of mite control achieved was lower than that reported in the studies mentioned above. Therefore, the trickling and cellulose methods seem to be more efficient ways of applying oxalic acid in hives.

Treatment 2, essential oils delivered in an ethanol-gelatin solution from absorbent pads, was the least efficacious of the formulations tested. The solvent (ethanol) was used to accelerate the release of essential oils from the pads. Perhaps the rate of release of the essential oils decreased after a quick evaporation of the ethanol, resulting in reduced varroacide efficacy of the oils tested. The average temperatures during the treatment periods were below 15 °C with very high humidity (>90%). It has been shown that low temperatures and high humidity decrease the amount of essential oils delivered in hives in the region where the experiments were conducted (Emsen et al. 2007), which may have happened after the ethanol evaporated from the pads.

In addition to oregano oil, clove oil and its major component eugenol have also demonstrated miticidal activity both under laboratory and colony conditions. Sammataro et al. (1998) found that varroa mites in Petri dishes exposed to the vapors of clove oil dissolved in olive oil killed 87.2% of the mites. In hives, Hoppe (1990) treated honey bee colonies with 10 mL of clove oil embedded in cardboard plaques and found low mortality rates of varroa mites (47%), similar to our results.

The varroacidal efficacy of oregano oil delivered with vaporizers in our study is the highest so far reported for this compound at the hive level. The miticidal activity of oregano oil is attributed to terpenes like carvacrol (Isman 2000). In our study, the oregano oil used contained 69% carvacrol, but the concentration of this component in the oil may vary from source to source (Isman 2000), which could affect the oil’s varroacidal efficacy, something to consider when this essential oil is used.

A few studies had previously tested oregano oil alone or in combination with other compounds in hives. Gal et al. (1992) used cardboard soaked with a 50% ethanol solution containing 10 or 16 mL of oregano oil and treated colonies under subtropical conditions. The average mite mortality was 82 and 91% for the two concentrations tested, respectively. In other studies, it was found that the application of a mixture of several oils, including oregano oil impregnated in cardboard strips, provided low mite control in temperate climates (Sammataro et al. 1998; Skinner et al. 2001). Finally, Romo-Chacón et al. (2016) applied oregano oil impregnated in cotton towels, finding 57–74% efficacy for varroa mite control. They also found residues of carvacrol in honey samples from their treated colonies that were below the 1.1 mg/kg taste threshold (Imdorf et al. 1999). We did not analyze samples of honey and wax for residues of oregano and clove oils from this study, but our group collected samples of honey and wax from a preliminary study of colonies treated with oregano oil and the levels of this compound in the samples were <0.16 mg/kg (Gashout et al. unpublished data), which agrees with the study of Romo-Chacón et al. (2016).

An advantage of using oregano oil in hives is that it is generally recognized as safe. In fact, oregano oil and derivatives of oregano are widely used in the food industry to prevent bacterial contamination because of its low toxicity for non-target organisms, particularly mammals, for being environmentally friendly and for its wide acceptance by the general public (Olivier 1997). Additionally, European Union regulations consider most essential oils and their components as drugs that do not need a maximal residue limit in food. Furthermore, oregano oil as potential varroacide has been found to be highly selective and with high lethal dose margin between host and parasite compared with tau-fluvalinate. This selectivity and high safety margin was demonstrated by Gashout and Guzman-Novoa (2009) in their study of the relative toxicity of this essential oil to *V. destructor* and honey bees.

The proportion of mites killed at different treatment periods was also associated with the miticide delivery system. About 65% of the total number of mites that died during the 4-week trial period, fell during the first 2 weeks from colonies treated with oregano oil delivered with electric vaporizers. However, only between 23 and 33% of the mites fell during this period with the other treatments, including the control. Furthermore, about 50% of the mites killed with essential oils delivered in pads fell during the last week of treatment as opposed to less than 9% in the case of essential oil released by vaporizers. The different patterns of weekly mite fall between treatments coincide with the predicted dynamics of *V. destructor* population calculated by computer simulations by Koeniger and Fuchs (1988). Under a scenario in which the effect of treatment is limited to a short period after application, mites that are not exposed to the miticide during this short time (such as those hidden in capped cells) survive. After the short-term miticide effect, mites leave cells, parasitize adult bees and invade brood cells again. Therefore, a short-term miticide effect cannot satisfactorily control *V. destructor* infestations until there is no brood in the colonies and mites infesting adult bees are exposed to the miticide. This may have happened in the case of the oregano and clove oils treatment released from pads as well as in the oxalic acid treatment. Conversely, under a scenario of continuous treatment at a sufficient dose over an extended period, mites are killed as they emerge from brood cells. In this case, it would be expected that a large proportion of the mites be killed within the first 2 weeks of treatment and very few of the ones remaining, during the last week of treatment. This seems to have happened with the oregano oil–vaporizer treatment. Early elimination of mites is critical because it results in longer bee lifespan and higher colony survival after winter (van Dooremalen et al. 2012).

The heat produced by electric vaporizers like those used in this study allow for continuous evaporation of volatile liquids. Therefore, this simple technology can be very helpful for continuously delivering essential oils in hives, particularly during seasons with low ambient temperatures like during fall in northern climates. Although the vaporizers could not sustain the rate of evaporation achieved during the first 2 weeks of treatment (2.11 ± 0.04 g/day), which declined almost four times during the last 2 weeks (0.55 ± 0.02 g/day), they continued providing a significant level of mite control, indicating that the release of 0.55 g of oregano oil per day provides adequate *V. destructor* control. The reduction in essential oil release was probably due to lower ambient and colony temperatures towards the end of treatments. The average ambient temperature during the first 2 weeks of treatments was 14.9 ± 0.79 versus 12.6 ± 1.06 °C during the last 2 weeks. Also, no brood was detected in colonies in the last 2 weeks of the treatment trials and thus, mites could not hide from the continuous exposure to all miticides when brood was not present.

We used 120 V AC power to operate the vaporizers. This is of course a limitation and is not practical for most beekeepers. However, the purpose of testing this delivery system was to demonstrate a proof of concept. That is, to show that a continuous release of a treatment can provide a high level of varroa mite control in hives. Our results support the notion that this seems to be the case, at least under the conditions and region within which we conducted the trials, and thus, the hypothesis tested cannot be rejected. Affordable vaporizers would need to be manufactured to make this concept practical and applicable. For example, similar vaporizers designed to work with rechargeable batteries or with energy from external solar panels could be alternatives for providing the necessary power for heating the vaporizers. The problem of decreasing evaporation rates in cold ambient temperatures could be addressed by supplying more vaporizers equipped with thermostats in each hive. If more evaporation would be required at lower temperatures, additional vaporizers could be turned on by a thermostat. Alternatively, other devices that are able to provide a continuous and constant rate of miticide release in hives might function with other mechanisms, but in any case, at this point, the assistance of engineers to design such devices using existing technology is warranted.

Bee mortality was higher in colonies treated with oxalic acid, although not significantly different than in the oregano oil–vaporizers and control colonies. Similarly, Giovenazzo and Dubreuil (2011) did not find higher bee mortality in oxalic acid treated colonies compared to other treatments in a similar climate to that where this study was conducted. However, mortality in the colonies treated with the mixture of oregano and clove oils, was about three times higher and significantly different than that observed in the oregano oil–vaporizers and control colonies. Previous studies by our group (Gashout and Guzman-Novoa 2009) found that the LD_50_s of clove and oregano oils were significantly lower than that of tau-fluvalinate for varroa mites, but were higher than those of the synthetic miticide for brood and adult bees. These results indicate that while being toxic to the mites, these essential oils were relatively safe for bees under laboratory conditions. However, Hoppe (1990) found in field trials, that when 10–15 mL of clove oil was applied in hives, bee mortality reached 27–50%, indicating high toxicity to the bees under field conditions. Due to discrepancy in results between laboratory and field settings, it seems reasonable to propose that further experiments are conducted to better assess bee mortality in colonies treated with oregano and clove oils embedded in pads, perhaps with a larger number of hives per treatment.

In conclusion, the results of this study show that a continuous release of natural miticides is important to achieve safe and high rates of varroa mite control in hives. They also show that oregano oil is effective for the control of *V. destructor* infestations in honey bee colonies. Future studies are warranted to confirm these results and to test different doses of oregano and other essential oils, as well as different devices designed to provide continuous release of miticides at a constant dose regardless of climatic conditions. Additionally, long-term studies are necessary to determine if treatments do not adversely affect bee health, colony development and productivity.
